# The Role of Obstructive Sleep Apnea in Vision-Threatening Diabetic Retinopathy—A National Register-Based Study

**DOI:** 10.3390/jpm13111529

**Published:** 2023-10-25

**Authors:** Shakoor Ba-Ali, Poul Jørgen Jennum, Adam Elias Brøndsted, Steffen Heegaard, Henrik Lund-Andersen

**Affiliations:** 1 Department of Ophthalmology, Rigshospitalet, Valdemar Hansens Vej 3, 2600 Glostrup, Denmark; adameliaz@gmail.com (A.E.B.); sthe@sund.ku.dk (S.H.); helan@regionh.dk (H.L.-A.); 2 Danish Centre for Sleep Medicine, Neurophysiology Clinic, Rigshospitalet, Valdemar Hansens Vej 3, 2600 Glostrup, Denmark

**Keywords:** obstructive sleep apnea, diabetic retinopathy, continuous positive airway pressure, hypoxia

## Abstract

Introduction: we investigated the association between OSA and vision-threatening diabetic retinopathy (VTDR). Methods: we used three nationwide registers to identify subjects with and without OSA and patients with type 1 (T1DM) or type 2 diabetes mellitus (T2DM). The Danish Civil Registration System was used to link OSA with diabetes diagnosis. The primary outcome was the occurrence of VTDR in diabetic patients with and without OSA. The secondary outcome was the prevalence of diabetes mellitus in patients with and without OSA. Results: we included 532,828 diabetic subjects comprising 13,279 patients with OSA (2.5%) and 519,549 without OSA (97.5%). Diabetic patients with OSA had a 57% lower risk of VTDR compared to diabetic patients without OSA (OR 0.43, 95% CI 0.38–0.50, *p* < 0.0001). Conclusions: our findings indicate that OSA is associated with a lower risk of VTDR. Since we did not adjust our outcomes for diabetes duration, hypertension control and hemoglobin A1c, future studies are needed to confirm our findings.

## 1. Introduction

Obstructive sleep apnea (OSA) is characterized by intermittent obstruction of the upper airway, leading to cessation of breathing (apnea) and/or reduced airflow (hypopnea), which last ≥10 s during sleep [1,2]. OSA is diagnosed using polysomnography (PSG), which is the golden standard. However, PSG requires in-laboratory measurements overnight, and it is therefore costly and resource demanding. Thus, the consensus is to perform cardiorespiratory monitoring (CRM) in an out-patient setting. The severity of OSA is described using an index called the apnea-hypopnea index (AHI), which is defined as the total number of apnea and hypopnea experienced per hour of sleep [3]. AHI < 5/h defines no OSA, AHI 5–15/h is mild OSA, AHI 15–30/h is moderate OSA, and AHI > 30/h is severe OSA. OSA occurs in approximately 3–50% of the general population, corresponding to nearly 1 billion people worldwide, with a predominance among obese, diabetic patients and males with increased age [4,5,6,7,8,9,10]. With an increasing obesity incidence and ever-growing elder population, it is plausible to expect a higher rate of OSA worldwide [11]. OSA is associated with high social and economic expenses; the American Academy of Sleep Medicine estimated the cost of diagnosing and treating OSA to be USD 12.4 billion in the USA in 2015 [12]. The economic burden of untreated OSA in US adults is even higher, and in 2015, was estimated to be approximately USD 149.6 billion [12].

OSA patients experience sleep fragmentation, snoring, fatigue, gasping, headache, poor concentration and excessive daytime sleepiness. Severe sleepiness during wake-time hours may slow reaction time, and OSA is shown to increase motor-vehicle accidents by 17% [13].

OSA is associated with cardiovascular diseases, metabolic syndrome, hypertension, depression and diabetes mellitus [14,15]. The prevalence of OSA in patients with type 1 diabetes (T1DM) ranges between 10% and 46% [16,17,18], whereas in patients with type 2 diabetes (T2DM) the range is 39–86% [19,20,21,22]. Due to repeated hypoxic events, an association between diabetic retinopathy (DR) and OSA has been suggested [4,23,24]. However, two recent meta-analyses have shown conflicting results [24,25]. Leong et al. could not find any compelling evidence to support the association between OSA and DR and suggested the need for larger studies [25]. In contrast Zhu et al. showed an increased risk (OR = 2.01, 95% CI = 1.49–2.72) of DR in patients with OSA [24]. Lately, a large Danish register-based study conducted by Grauslund et al., including 153,238 diabetic patients and 746,148 non-diabetic controls, showed a decreased risk (HR, 0.83; 95% CI, 0.74–0.92) of developing DR in diabetic patients with OSA [26]. Later findings by Grauslund et al. contradict the conventional pathophysiologic factors in the development of DR including hypoxia, oxidative stress and inflammation [27]. The retina is dependent on the oxygen diffusion from intraocular blood supply. Diabetic patients suffer from high rates of vascular abnormalities, and it is likely that the combined effect of nocturnal glucose dysregulation, impaired blood supply and apnea-related hypoxia aggravate the retinal anoxia, leading to further retinal damage. An inverse relationship between OSA and DR seems controversial, and further investigation is needed to elucidate the role of OSA-mediated hypoxia in developing DR. 

We investigated the risk of developing vision-threatening diabetic retinopathy (VTDR) in diabetic patients with and without sleep apnea. 

## 2. Materials and Methods

### 2.1. Subjects

We included diabetic patients with OSA and diabetic patients without OSA. Our primary outcome is VTDR, and to find the prevalence of VTDR, we further grouped the subjects into diabetic patients with PDR and diabetic patients without DR among both OSA and non-OSA groups. We only included those patients with PDR who were either treated with retinal photocoagulation or anti-vascular endothelial growth factor inhibitor (anti-VEGF). Thus, PDR is defined as PRP and anti-VEGF. Since many diabetic patients have DR and DME simultaneously, and patients with DME receive anti-VEGF, our study population included both patients with PDR and DME. However, patients with PDR constituted most of our study population, because anti-VEGF was not approved until 2007 and the index date was 1980 to 2014. 

We also looked at the opposite model, i.e., we found all patients with OSA and investigated the proportion of those with diabetes and their age- and sex-matched controls. 

### 2.2. Data Extraction

The Danish Civil Registration System (CRS) enables scientists to conduct large, national register-based studies by linking the CRS to other registers and biobanks using a unique identification number called a Central Personal Registration Number, assigned to each inhabitant in Denmark [28]. The CRS contains demographics, migration and vital statistics. Registration to the CRS has been conducted electronically since 1968 and non-electronically since 1924 [28]. The CRS has been updated on a daily basis since 1989 [28]. The other registers used in this study include the Danish National Patient Register (DNPR) and the Danish National Prescription Registry (DNMR). The DNPR has existed since 1977 and provides information on hospital admission type and date, clinical procedures and diagnosis according to the International Classification of Diseases codes (ICD 8th and 10th revision) for all patients discharged from Danish hospitals. 

DNMR records detail information on the prescription, patient and prescriber on an individual level for all prescriptions dispensed from pharmacies in the Danish community using the Anatomical Therapeutic Chemical (ATC) Classification System. 

In the current study, we used the DNPR to identify patients with T1DM (DE10) or T2DM (DE11) registered from 1980 to 2014 and patients with OSA (G473) with the index date between 1994 and 2014, matched by age and sex. We also looked at the prevalence of diabetes among patients with OSA and their age- and sex-matched controls with an index date between 1994 and 2016. The index date is the first time the patient appears in the register. To include diabetic patients without hospital contacts, we used the ATC codes for insulin (A10A) and non-insulin diabetes medication (A10B) in the DNMR. To identify patients with PDR and DME, we used the Health Care Classification System (SKS) for retinal photocoagulation laser including pan-retinal photocoagulation and local retinal photocoagulation (KCKC10 and KCKC15) and anti-VEGF (BOHJ19B) in DNPR. 

### 2.3. Statistics 

Data are presented as numbers, percentages or mean ± standard deviation (SD). A multivariate logistic regression model was used to calculate the odds ratio (OR) and 95% confidence interval (95% CI) for VTDR in diabetes patients with and without OSA. The logistic regression model adjusts for age, sex and marital status.

## 3. Results

In total, we included 532,828 patients with T1DM or T2DM, consisting of 13,279 patients with a diagnosis of OSA (2.5%) and 519,549 diabetic patients without OSA (97.5%), Table 1. Patients with OSA were predominantly male (76%) with an average age of 56 years and 69% of them lived with their spouse. The prevalence of VTDR was 1.6% higher in diabetic patients without OSA compared to diabetic patients with OSA. The risk of developing VTDR was 57% lower in diabetic patients with OSA compared to diabetics without OSA (*p* < 0.0001), Table 1. Male diabetics had a 26% higher risk of developing PDR/ DME compared to females (*p* < 0.0001), and diabetic patients living with their spouse had a 13% higher risk of having VTDR (*p* < 0.0001). Diabetic patients without OSA were six years older compared to diabetic patients with OSA (*p* < 0.0001). 

We investigated the inverse situation, hence the prevalence of diabetes among patients with and without OSA. The proportion of diabetes diagnoses was twice as high among patients with OSA (9.5%) compared to the prevalence of diabetes in non-OSA controls (4.4%), Table 2.

## 4. Discussion

The main findings of this national register-based study were that OSA is associated with a lower risk of VTDR. In addition, our results confirm previous study results regarding the fact that diabetes mellitus is more prevalent among patients with OSA compared to non-OSA individuals. 

The pathophysiology of OSA is decreased muscle tonus of the pharynx during sleep, leading to intermittent collapse of the oropharynx, obstruction of airflow into the lungs and consequently, decreased respiratory ventilation. The condition is associated with daytime sleepiness, snoring, frequent microarousals and decreased reaction time. On a cellular level, the consequences of apnea, hypopnea and microarousals are hypoxia, systemic inflammation, oxidative stress and sympathetic nerve system activation [29]. Increased sympathetic nerve system activity leads to a high level of norepinephrine, which results in increased lipolysis, glycogenolysis and insulin resistance [30]. OSA is shown to be associated with glucose intolerance and T2DM [31]. Our results agree with these previous findings, showing a higher prevalence of diabetes among OSA patients [32]. Whether OSA causes diabetes and vice versa is still not established firmly, but both disease entities have many common risk factors, importantly, obesity. 

So far, the VTDR in patients with OSA is contentious, since the pathophysiologic consequences of OSA are hypoxia, oxidative stress and inflammation, which in turn are assumed to be important pathophysiologic mechanisms in the etiology of VTDR [27,33]. In the published literature, a significant amount of research indicates a positive relationship between OSA and DR. Zhu et al. published a meta-analysis on this topic including six studies with a total of 608 diabetic OSA patients and 484 diabetic patients without OSA [24]. Their pooled results showed an increased risk of DR (OR; 2.01, 95% CI; 1.49–2.72) in patients with OSA [24]. In another review study, comprising 16 studies with 2636 patients with T2DM, Leon et al. reported an association between OSA and advanced DR in 10 out of 16 studies [25]. Most of the studies included in the two studies had retrospective designs, using heterogenous outcomes for OSA [26]. It is worth noting that the majority of the studies use the apnea-hypopnea index (AHI) as a predictor for development of DR; however, as suggested by Leon et al., nocturnal hypoxemia or apnea-related arousal during sleep maybe better predictors for the development of DR in OSA patients [25]. Leon et al. also found several methodological pitfalls in previous studies. 

In contrast to the above-mentioned studies, Grauslund et al. recently conducted a large register-based study, including 153,238 diabetic patients and 746,148 non-diabetic controls, showing that diabetic patients with OSA have a 17% decreased risk of developing DR over a 5-year period compared to diabetics without OSA [26]. 

The study by Grauslund et al. is the largest on this topic, and their findings are in agreement with our study [26]. Although our study is overall based on the same population as used in the study by Grauslund et al., there is an important difference between the two studies, namely that we included patients with PDR and DME, whereas Grauslund et al. have a relatively small sample with PDR [26]. The advantage of the study by Grauslund et al. is that they adjusted their results for diabetes duration, antihypertensive treatments and diabetes controls. Progression to VTDR is associated with diabetes duration, diabetes control and hypertension. Given the size of our study population (532,828 diabetic patients), we believe that the large study population compensates for those confounders, since there is a high chance that these confounders are equally distributed in the different groups.

The retina is one of the highest energy-consuming tissues, and due to OSA-related intermittent nocturnal hypoxemia combined with diabetes-induced general atherosclerosis, one may assume the likelihood of further retinal hypoxic stress and consequently, a higher risk of DR [31,34,35,36]. Surprisingly, we found a lower risk of developing VTDR in diabetic patients with OSA. One explanation for the lower occurrence of PDR could be that the retinas of diabetic patients may adapt to the decreased level of O_2_. This ability of diabetic retinas to adapt to metabolic stress has previously been shown in diabetic patients under oral glucose-induced acute hyperglycemia [37]. Another explanation for the lower risk of VTDR in OSA patients could be that they might have received better diabetes care to avoid complications due to a higher awareness among diabetes clinicians of the higher risk profile of diabetic patients with OSA. For example, it is highly likely that diabetic patients with OSA have received continuous positive airway pressure (CPAP) treatment. In Denmark, CPAP treatment is indicated in patients diagnosed with moderate to severe OSA. CPAP treatment is very effective in restoring the airway and prevents the airway from re-collapsing. However, CPAP treatment is associated with decreased compliance, which is reported in up to 83% of patients [38]. The decreased compliance can be due to the fact, that CPAP treatments include wearing a mask, which is not always tolerated by the patients. In addition, CPAP treatment implies the flow of oxygen from the mask through the nose and mouth, which can cause a dry, stuffy nose. Nevertheless, when diabetic patients adhere to CPAP treatment, it reduces the incidence of diabetic retinopathy. This has been confirmed in a retrospective study, showing a 46% lower prevalence of DR in diabetic OSA patients compliant with CPAP use compared to non-compliant patients [39]. 

In this study the prevalence of OSA among diabetic patients was only 2.5%. This prevalence is markedly lower than previously published for both type 1 and type 2 diabetes (10–46% in T1DM, 39–86% in type 2 T2DM). It is apparent that either the estimates of OSA in the diabetic population are grossly overestimated, or that it is very greatly underdiagnosed/undertreated in this system. The prevalence of OSA was 5.8% in T2DM in the study conducted by Grauslund et al., which is slightly higher than the prevalence of OSA in our study. The slightly higher prevalence of OSA in Grauslund’s study might be attributed to the fact that OSA is more prevalent in T2DM [26], and since we included both T1DM and T2DM, the pooled prevalence is slightly lower in our study. As both ourselves and Grauslund find a lower prevalence of OSA in diabetes patients compared to previous studies and our studies are the largest on this topic, we might assume that previous studies could have overestimated the prevalence of OSA in diabetes patients.

## 5. Limitations

OSA is associated with a high mortality rate, hence it is highly likely that patients with OSA will die at an earlier age, even before they develop a severe degree of DR [40,41]. This is supported by our data, showing that diabetic patients with OSA are significantly younger than patients without OSA (Table 1). Thus, a high mortality rate in diabetic patients with OSA is an important confounder, which we did not adjust for. On the other hand, CPAP treatment also decreases the risk of strokes and heart attacks [42]. Hence, one may assume that CPAP adherence also reduces the risk of mortality; thus diabetic patients with OSA may live longer due to better care including CPAP treatment compared to diabetic patients without OSA. Thus, this area needs further research to clarify the role of CPAP treatment in the risk of developing retinopathy among patients with and without OSA. 

Another important limitation is the fact that OSA is underdiagnosed because diagnosis is time consuming and requires special equipment, and certified staff are required to screen for OSA. 

We did not adjust our outcomes for HbA1c, diabetes duration and hypertension control. Given our large study population and that the vast majority of our study population includes diabetic patients with a history of pan-retinal photocoagulation, we can assume that diabetes duration and hypertension control might be similar in diabetic patients with and without OSA.

We identified diabetes patients based on medication prescriptions, which may also have included patients with pre-diabetes, as individuals with pre-diabetes are prescribed metformin. We did not extract data on the obesity status; hence, no multi-variable adjustments were made for confounding factors such as obesity.

## 6. Conclusions

In conclusion, this paper reports that OSA does not increase the risk of VTDR in diabetic patients. Since we do not adjust our outcomes for diabetes duration, HbA1c and hypertension control, future studies are needed in which adjustments for these parameters are performed.

## Figures and Tables

**Table 1:** Using logistic regression to assess the risk of developing proliferative diabetic retinopathy (PDR) or diabetic macular edema (DME) in diabetes patients with and without obstructive sleep apnea (OSA).

	Without OSA N (%)	With OSA N (%)	OR (95% CI) for PDR	*p* Value
Patients with diabetes, index date 1980–2014	519,549 (97.5%)	13,279 (2.5%)	N total: 532,828	
VTDR	17,210 (3.3%)	231 (1.7%)	0.434 (0.38; 0.50)	<0.0001
Descriptive at index date				
Female	249,687 (48.1%)	3234 (24.4%)	0.743 (0.72; 0.77)	<0.0001
Male	269,862 (51.9%)	10,045 (75.6%)		
Age (mean ± SD)	61.6 (±17.6)	55.6 (±12.5)	0.975 (0.97; 0.98)	<0.0001
No spouse *	211,245 (40.7%)	4171 (31.4%)		
Spouse *	308,304 (59.3%)	9108 (68.6%)	1.134 (1.10; 1.17)	<0.0001

* For children, parents’ marital status is used. DME: diabetic macular edema. OR: odds ratio. OSA: obstructive sleep apnea. PDR: proliferative diabetic retinopathy.

**Table 2:** Prevalence of diabetes (including both type 1 and type 2 diabetes mellitus) in patients with obstructive sleep apnea (OSA) and sex- and age-matched non-OSA controls.

	Controls N (%)	Patients with OSA N (%)
Diabetes, index date 1994–2014	12,557 (4.4%)	6744 (9.5%)
Not diabetes	272,423 (95.6%)	64,502 (90.5%)
All	284,480 (100%)	71,246 (100%)

## Data Availability

Additional data will be available upon further request.
